# The Acidic Microenvironment: Is It a Phenotype of All Cancers? A Focus on Multiple Myeloma and Some Analogies with Diabetes Mellitus

**DOI:** 10.3390/cancers12113226

**Published:** 2020-11-02

**Authors:** Stefano Fais, Yoshinori Marunaka

**Affiliations:** 1 Department of Oncology and Molecular Medicine, Istituto Superiore di Sanità (National Institute of Health), 00161 Rome, Italy; 2 Research Institute for Clinical Physiology, Kyoto Industrial Health Association, Kyoto 604-8472, Japan; 3 Research Center for Drug Discovery and Pharmaceutical Development Science, Research Organization of Science and Technology, Ritsumeikan University, Kusatsu 525-8577, Japan; 4 Department of Molecular Cell Physiology, Kyoto Prefectural University of Medicine Graduate School of Medical Science, Kyoto 602-8566, Japan

**Keywords:** multiple myeloma, therapy, acidity, proton transports inhibition, type diabetes

## Abstract

**Simple Summary:**

Multiple myeloma (MM) is a hematological malignancy characterized by an abnormal clone of plasma cells in the bone marrow. Currently, both the disease progression and its therapy are too often followed by a series of complications and the standard treatment for MM is aimed at improving the quality of life and to prolong progression-free survival (PFS), and overall survival (OS) of patients. This review looks at MM therapies from a different sight. It starts from a clear scientific background, suggesting that many malignant tumors have some common phenotype that isolates the tumor from the rest of the body. Between these phenotypes, extracellular acidity exerts a key role and data collected in the last two decades support the use of anti-acidic drugs in the treatment of cancers, including MM. Lastly, as many cancers, MM has some similarities with type II diabetes mellitus (DM) in the mechanism leading to the extracellular acidity, supporting the use of both a wide panel of proton transporters inhibitors and probably also of some anti-DM drugs in the treatment of both MM and DM.

**Abstract:**

Multiple myeloma (MM) is a hematological malignancy with a poor prognosis while with a long and progressive outcome. To date, the therapeutic options are restricted to few drugs, including thalidomide or its derivates and autologous transplantation including stem-cell transplantation. More recently, the use of both proteasome inhibitors and monoclonal antibodies have been included in MM therapy, but the clinical results are still under evaluation. Unfortunately, death rates (within the 5-year overall survival rates) are still very high (45%), with no relevant improvement over the past 10 years. Here, we discuss data supporting a new therapeutic approach against MM, based on a common phenotype of tumor malignancies, which is the acidic microenvironment. Extracellular acidity drastically reduces the efficacy of both anti-tumor drugs and the immune reaction against tumors. Pre-clinical data have shown that anti-acidic drugs, such as proton pump inhibitors (PPIs), have a potent cytotoxic effect against human MM cells, thus supporting their use in the treatment of this malignancy. Here, we discuss also similarities between MM and type II diabetes mellitus (DM) with high risk of developing MM, suggesting that both anti-diabetic drugs and a hypocaloric diet may help in curing MM patients.

## 1. Introduction

The key role of the tumor microenvironment in both cancer generation and progression has been investigated during the last two decades. Actually, cancer evolution is dictated from the continuous changes due to microenvironmental selection forces that in turn induce a sort of Darwinian selection, generating phenotypes that differentiate cancer cells from the rest of the body. These phenotypes represent a selective advantage for tumors in respect to normal or more differentiated cells. In fact, tumors can differentiate from the rest of the body inasmuch as they grow in a peculiar ecosystem, as composed by the cancer cells, stromal cells, vasculature, extracellular matrix, and the chemical milieu, consisting of some variables including the pH and the oxygen tension. Moreover, during growth and progression, the tumor ecosystem shows considerable plasticity inasmuch as cancer cells continuously shape their microenvironment in response to both the body reaction and the therapeutic strategies, including both chemotherapy, targeted therapies, surgery, and radiotherapy; this occurs in an adaptive way, which may be summarized as “the fittest survive”. Low supply of oxygen (hypoxia), nutrient deprivation, products of tumor metabolism, and increased extracellular protons (H^+^) are all microenvironmental phenotypes putting tumors under a selective pressure. This microenvironmental pressure on one end selects cells naturally armed to survive in these so unfavorable conditions; on the other end induces cancer cells to further adapt to the tumor microenvironment. Moreover, vast areas of the tumor mass periodically undergo either apoptosis or necrosis, which is a frequent finding during the prognostic follow-up of cancer patients. The result of this continuous remodeling of tumor fitness generates a sort of competition between cells to survive and this is not granted for the cells more armed to survive in this very hostile microenvironment, inasmuch as each time a massive necrotic event occurs within a tumor mass, this selection re-start from the beginning. In this way, the tumor isolates itself from the rest of the body, thus contributing to the tumor escape from the anti-tumor response, but also the resistance to many of the existing therapies. The extracellular acidity is probably the most important microenvironmental factor inducing this sort of continuous remodeling. Extracellular acidity is involved in: (i) Resistance to therapy; (ii) Tumor immune escape; (iii) Increase tumor progression; (iv) the metastatic behavior; (v) The exosome release by cancer cells and tumors. Since extracellular acidity is a common feature of virtually all cancers, it appears conceivable that an anti-acidic treatment may well represent a very important and promising new approach against cancers, both in combination with existing therapies and a first line therapy.

Some clinical reports have shown that diabetes mellitus (DM) is associated and predisposes to cancer conditions. In fact, metabolic acidosis is a common feature in DM and peripheral tissue acidity is involved in a reduced affinity of insulin receptors for insulin. Moreover, DM patients too often develop cancers in their life. This suggests that an anti-acidic approach may be considered also a preventive measure against cancer.

## 2. Multiple Myeloma (MM): More Shadows than Lights

MM is a hardly curable disease with unfortunately more than 60,000 deaths annually. MM represents 10% of all hematological malignancies [1,2,3]. From the clinical point of view, MM derives from the expansion of malignant plasma cells, very often in monoclonal way, occurring into the bone marrow, and with a progressive depletion of normal cells and to a complex clinical picture with hyperglobulinemia, hyperviscosity, coagulation disorders, and organ damage secondary to k chains deposition with often amiloidosis. MM is resistant to virtually all the existing therapies, including chemotherapy protocols, but also the new targeting therapies (e.g., bortezomib, proteasome inhibitor (PI), thalidomide, lenalidomide, and immunomodulatory drugs [4,5]). In addition, this resistance tends to occur very rapidly, thus leading to a death rate (5-year overall survival rate) ranging between 40 and 50%, with no relevant improvement over the past 10 years [6]. It is therefore mandatory to investigate new and effective therapies against MM, inasmuch as an effective therapy remains an unmet clinical need.

## 3. The Acidic Microenvironment, a Phenotype Common to All Cancers and Proton Pump Inhibitors (PPIs)

A hallmark common to virtually all malignant tumors is the extracellular low pH (very often associated to an alkaline cytoplasmic pH, the so-called pH gradient reversal) [7]. The kinetics of this pH gradient reversal of malignant tumors begins with the so called “Warburg effect”. It consists of preferential metabolism aimed at sugar fermentation, with extracellular lactate accumulation, resulting in proton (H^+^) accumulation in the extracellular spaces [8]. To survive in this very hostile microenvironment, tumor cells have to actively express a series of functions that protect them from some intracellular events. For instance, quick cytosolic acidification may lead to cell death in some instances. One of the most effective strategies to counteract this condition is represented by the overexpression of proton-extruding transporters at different classes, including proton pumps and ion exchangers. Between these cellular pumps, vacuolar-type (V-type) H^+^-ATPases exert a key role, with a double function actually, to pump protons into the internal vesicles and to eliminate protons into the extracellular space, thus fueling the extracellular acidity. The extracellular acidity represents clearly the most effective condition contributing to all the known malignant tumor activities, including proliferation, invasion, metastasis, and drug resistance [9,10,11]. PPIs are a class of inhibitors of ATPases, including V-type H^+^-ATPases and H^+^/K^+^-ATPases. PPIs include a series of commercially available molecules that are used worldwide as anti-acidic drugs. They include Omeprazole, Esomeprazole, Rabeprazole, Pantoprazole and Lansoprazole [12,13]. All these molecules are administered as prodrugs which need an acidic environment in order to be transformed in the active molecule, tetracyclic sulfonamide, inhibiting V-type H^+^-ATPases and H^+^/K^+^-ATPases [14]. The paradox is that while the great majority of the drugs (that are weak bases) are protonated, and thus blocked once, got to the tumor microenvironment, PPIs in the same condition are transformed into the effective molecule. This approach has led to the scientific evidence that PPIs on one hand allow chemotherapeutics to be largely more effective against the same resistant tumor cells and tumors, on the other hand, by blocking a survival function, i.e., proton pumps, PPIs have shown a clear anti-tumor effect, with cytotoxicity [15,16,17,18,19]. Notably, the chemo-sensitive and the anti-tumor effects of PPI were always consistent with an in vitro and in vivo reduction of microenvironmental acidity [20,21,22,23]. Experimental evidence has shown that PPIs are effective against a variety of cancers regardless of the tumor histology. Moreover, both pre-clinical and clinical evidence have shown clearly that PPIs are not toxic, or in any event do not increase pre-existing toxicity [11,16,17,24,25,26,27,28,29]. PPIs belong to a family of generic drugs, but they have some important differences in term of both pharmacodynamics and bioavailability. In fact, one set of published data have shown that Lansoprazole had the greatest anti-tumor effect, when compared to the other PPIs [19]. This would be probably due to the lipophilic nature of Lansoprazole as compared to the other PPIs [19]; although of course, it needs further investigation to confirm this only partially demonstrated hypothesis [19]. However, a series of ion exchangers inhibitors have proven to represent a very promising weapon against cancer [10] and therapies based on combination between them have been shown to be highly effective in pre-clinical settings [30]. The data on treatment with specific inhibitors of proton-extruding transporters, solid pre-clinical data have shown that systemic alkalization is highly effective in both preventing [31] and treating cancer [32].

## 4. MM, Acidity, and PPI

In a preliminary study, we showed that PPI induced cytotoxicity against hematologic malignancies-derived cells in both in vitro (B cell lymphomas-derived cell lines) and ex vivo (bone marrow blasts from acute lymphoblastic leukemia (ALL) children) [17]. In the same study, we also showed that the PPI-induced cell death was caspase-independent and was due to an early intracellular accumulation of both reactive oxygen species (ROS) and H^+^. On the basis of these results, we performed a comparable study using MM cells. The published data showed that the PPI Lansoprazole had a clear inhibitory effect on the growth of different human MM cell lines. Further, investigation showed that the inhibition on MM cells proliferation was rather due to apoptosis but not primary necrosis [33]. In the same study, it has been shown that MM cells died through an atypical apoptosis. All in all, Lansoprazole showed an anti-proliferative effect on MM cells via an atypical apoptosis. The caspase-independent apoptotic-like cell death shown in MM cell lines was consistent with the results obtained from other hematological malignancies [17]. Both investigations showed that treatment with pan-caspase inhibitor z-VAD-fmk in either MM cells or B-cell tumors-deriving cells was not able to inhibit Lansoprazole-induced cell death. However, this was not true in T cell derived cell lines such as Jurkat cells, where caspase inhibitors entirely inhibited the PPI-induced apoptosis [34]. Notably, we know that T cells undergo apoptosis via a caspase pathway, suggesting that PPIs induce different apoptotic pathways in human tumors depending on the different cell lineages. In supporting this hypothesis, we have previously shown that PPIs induce a caspase-dependent cell death in human melanoma cell lines [18]. However, we have also shown that the PPI-dependent cell death passes through both a quick cytosolic acidification and a ROS accumulation within treated cells [17,18]. A further study has shown that the PPI action induces a reduction of ATP consuming, thus supporting a clear inhibition of ATPases activity. Moreover, in PPI-treated cells, the reduced acidification occurs in both the intracellular acidic organelles and the extracellular spaces; this result was highly consistent with the proposed mechanisms underlying Lansoprazole action (the inhibition of V-type H^+^-ATPases) [15]. In different models of human tumor cells, we have also shown that PPI may be active at lower doses, and this is particularly true for Lansoprazole, thus giving a chance to start treatment with doses that may reduce possible systemic side effects [16]. In fact, some recent clinical reports have shown potential toxicity of PPI at the gastrointestinal level, particularly in patients treated with high doses of Lansoprazole [10,24,25,26,28]. However, the great news is that PPIs, in particular Lansoprazole, may be successfully used in the treatment of MM patients, alone or in combination with other drugs, in the first line as well as the second, third, or fourth lines of treatments, without having shown significant side effects, actually. Lansoprazole has been shown to be used as a daily treatment for a long time without evidence of side effects, and this is of paramount importance inasmuch as it is the PPI with the longest action, probably due to its lipophilic nature [10,24,25,26,28]. Lastly, we recognize also that PPIs increase the effectiveness of adoptive immunotherapies as well as the natural anti-tumor immune reaction, and this may be true for MM as well [35,36].

## 5. Mechanisms Causing Acid Microenvironments in MM Cells

**Figure 1:**
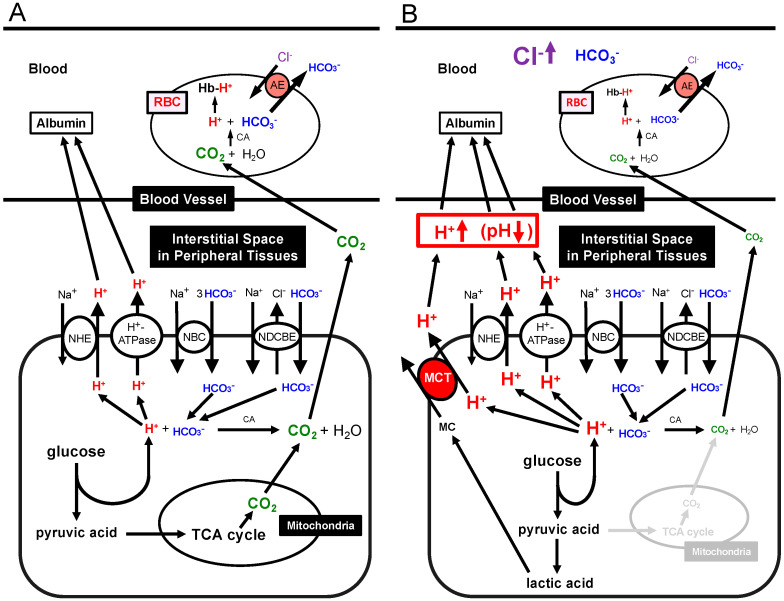
Production of H^+^ and CO_2_, and transporting systems of H^+^ and CO_2_ in peripheral tissues. (**A**) Production of H^+^ and CO_2_, and transporting systems of H^+^ and CO_2_ in peripheral tissues with ‘normal’ mitochondrial function: the glycolysis process produces H^+^ and TCA cycle generates CO_2_. (**B**) Production of H^+^ and lactic acid, and transporting systems of H^+^ and lactic acid in peripheral tissues with ‘dysfunction’ of mitochondria such as MM and DM cells. Much more amounts of H^+^ are produced via glycolysis in a case of mitochondrial dysfunction, MM and DM cells in order to produce the same amount of ATP as that with normal mitochondrial function. In a case of mitochondrial dysfunction such as MM and DM cells, TCA cycle has no or little function, thus the required amount of ATP is mainly generated via glycolysis, leading to production of much more amounts of H^+^ and lactic acid than that of the normal mitochondrial function. Modified from Figure 3 in Int. J. Mol. Sci. 2018, 19, 3244 [48].

As mentioned above, MM cells may undergo microenvironments acidification [7,8]. Indeed, multiple myeloma may acidify its microenvironment through mechanisms that are common to all malignancies. Here, we describe the mechanism that tumor cells, including MM cells, may use to acidify their microenvironment. It is well known that malignant tumor cells usually obtain energy (ATP) via the glycolytic pathway even under oxygen available conditions, the so called ‘Warburg effect’ [32,37]. This feature has been recognized as one of the key characteristics of malignant tumor cells, including MM [32,37,38,39]. The glycolysis-dominant metabolism produces a large amount of lactic acids and protons, leading to an early intracellular acidic condition, which induces a marked perturbation in virtually all cellular functions, in particular, in the enzymatic activities. To prevent the disturbance of the cellular function due to the intracellular acidity, malignant tumor cells have to keep normal intracellular pH by excreting a large amount of lactic acids and protons into the extracellular space. For the excretion of lactic acid and protons, malignant tumor cells including MM cells express various types of ion transporters such as H^+^-ATPase (pump), Na^+^/H^+^ exchanger (NHE), and/or monocarboxylate transporters (MCT) (e.g., MCT1, MCT4), which participate in the maintenance of a controlled intracellular pH (Figure 1) [32,37,38,39]. All in all, malignant tumor cells in order to avoid intracellular acidification overexpress a series of proton transporters (mostly H^+^-ATPase, NHE, and MCT) that pump H+ outside the cells (Figure 1). The expression of MCT1 and MCT4 correlates with the expression of the transmembrane glycoprotein CD147, which is overexpressed in MM cells [40]. MCTs overexpressed in MM cells transport a large amount of lactic acids and protons to the extracellular cells (Figure 1) [40,41,42,43]. Thus, overexpression of MCTs in the plasma membrane of multiple myeloma cells associated with elevated activity of MCTs is one of the key factors keeping normal intracellular pH condition for maintenance of MM cell functions (Figure 1). Indeed, siRNA-induced downregulation of MCT1 decreases proliferation of human MM cells associated with downregulated expression of CD147, decreased lactate extrusion, and increased extracellular pH [39]. Some studies report that patients suffering from MM show lactic acidosis [44,45,46,47].

Latic acidosis is categorized to (1) Type A lactic acidosis and (2) Type B lactic acidosis. Type A lactic acidosis is caused by anaerobic metabolism due to hypoxia. On the one hand, type B lactic acidosis shows anaerobic metabolism even under tissue normoxemia conditions in MM [44]. The acidic microenvironment mentioned above is also observed in DM. In the next section, we review mechanisms underlying tissue acidification that may be in common between DM and MM.

## 6. Acidity Is a Common Pathway Between MM and DM

In expanding what we have described in the above paragraphs, here we compare mechanisms regulating pH homeostasis in normal cells, with functional mitochondria under normoxemia conditions, and to what actually occurs in DM and MM cells, with dysfunctional mitochondria under normoxemia or hypoxemia conditions (Figure 1). In normal cells with functional mitochondria under normoxemia conditions, ATP is mainly synthesized in mitochondria, with only small amounts of H^+^ release into the intracellular spaces (Figure 1A). In MM and DM cells with dysfunctional mitochondria under normoxemia or hypoxemia conditions, large amounts of H^+^ are produced and released into the intracellular spaces (Figure 1B). This leads to the development of mechanisms that eliminate protons into the extracellular spaces, and, as a consequence, an acidification of the interstitial fluids; the major mechanisms underlying this condition in MM and DM cells are a secondary mitochondria dysfunction and overexpression of proton-extruding transporters (Figure 1B).

DM patients affected by metabolic syndrome (i.e., hyperglycemia, hyperinsulinemia, and dyslipidemia) are under high risk of developing various types of cancers including liver, pancreas, colon, rectum, breast, bladder cancers, and MM as well [37]. DM cells show metabolisms similar to MM cells: i.e., DM cells produce energy (ATP) mainly via glycolysis due to mitochondrial dysfunction even under aerobic conditions [37,48]. The glycolysis-based metabolic process occurring in DM due to mitochondrial dysfunction produces much larger amounts of protons compared to normal cells, showing normal mitochondrial function efficiently generating ATP via TCA cycle [37,48]. Protons are generated from organic acids, including lactate, as metabolites produced in living cells in the ATP-dependent processes [32,37,48,49,50,51,52,53,54]. One of the major organic acids producing protons in DM is lactic acid (lactate^−^/H^+^) [48,49,50], which is the final production of glycolytic metabolism of glucose and glycogen under anaerobic or mitochondria-dysfunctional conditions in cellular models, such as myocytes and adipocytes. The same condition is spontaneously observed in both MM and DM cells [37,55,56,57,58].

As mentioned above, MM shows lactic acidosis [44,45,46,47] and lactic acid also primarily plays an important role in extracellular acidification of DM cells. The pKa of lactic acid is ~3.8 at 37 °C [59], meaning that the lactic acid produced in metabolic cells exists as its ionized form (lactate anion (lactate^−^) + proton (H^+^)) under physiological conditions, ~pH 7.4 of the extracellular fluid and ~pH 7.2~pH 7.6 of the intracellular fluid. Thus, the intracellular fluid pH is decreased under conditions of poor oxygen supply or mitochondrial dysfunction associated with excess glycolysis occurring in metabolic disorders such as MM and DM [32,37]. In addition, ketone bodies are other major sources of protons pathologically produced from fatty acids in the liver when pancreatic beta-cells fail to secrete insulin or insulin resistance appears, while ketone bodies are physiologically produced from fatty acids as a physiological response to prolonged exercise or at reduced carbohydrate intake [48,60,61,62,63]. Local metabolic disorders occurring around MM cells would not primarily produce ketone bodies, as we can observe under DM complications; in fact, the production of ketone bodies always results in severe acidic microenvironments. β-hydroxybutyric acid (β-hydroxybutyrate^−^/H^+^) is one of major ketone bodies [60,62,63] and exists as its ionized form, since the pKa of β-hydroxybutyrate is ~4.8 in living bodies [64]. Thus, in normal condition, a balance does exist intracellularly between anions (β-hydroxybutyrate^−^) and protons (H^+^), with a pH ranging between 7.2 and 7.6) [32,37,48,61,62]. When β-hydroxybutyric acid is produced in hepatocytes under conditions of poor oxygen supply, including mitochondrial dysfunction and excess glycolysis, as it occurs in MM complicated with DM, it may lead to a reduced intracellular pH [32,37,48,61]. Metformin has been developed as a DM treatment drug for reduction of blood glucose levels by inhibiting gluconeogenesis in the liver [64]. In some cases, DM patients taking metformin show ketoacidosis via generation of ketone bodies such as β-hydroxybutyric acid due to metformin-caused insufficiency of glucose availability [64]. This means that regardless of the causes, MM patients complicated with severe DM patients would show ketoacidosis by generating ketone bodies such as β-hydroxybutyric acid due to insufficiency of glucose availability. This should oblige to pay attention to the occurrence of acidic microenvironments in MM patients complicated with DM. In summary, metabolic disorders may occur in both MM and DM, with insufficiency of glucose availability, and an increased production of acids such as lactic acids and β-hydroxybutyric acid, that directly contribute to the extracellular acidification. Moreover, disorders such as MM with DM may lead to either systemic acidosis or microenvironmental acidification, with a total derangement of pH homeostasis at both tissue and systemic levels (see details in the following section).

### 6.1. Preventing Systems from Lowered pH of the Intracellular Fluid in MM and DM

Acids such as lactic acid and β-hydroxybutyric acid, produced in metabolic cells, myocytes, adipocytes, and hepatocytes, lower the intracellular pH due to their pKa of ~3.8~4.8 [32,37,48,49,61,62,63,64,65]. The lowered pH of intracellular fluids disturbs cellular functions based on enzymatic activities [32,37,48,61,66,67]. Therefore, cells need to field all their strategies with the purpose of preventing the lowering pH in the intracellular fluid, and this occurs in a series of cellular models, such as myocytes, adipocytes, and hepatocytes, but in MM cells as well. Two major systems participate in the attempts of the cells to prevent from intracellular acidification: (1) the pH buffering system in the intracellular fluid and (2) the extrusion system of acids (protons) from the intracellular fluid to the extracellular space.

### 6.2. pH Buffering Systems in the Intracellular Fluid in MM and DM

The pH of intracellular fluids of living cells is generally maintained at 7.2~7.6 by various systems such as: (i) the protein–proton binding pH buffering system, (ii) the bicarbonate–carbonate (HCO_3_^−^−CO_2_) and phosphoric acids (H_3_PO_4_)-mediated buffering systems, and (iii) the proton-transporting system [48,68,69]: especially in MM and other types of malignant tumors, the intracellular pH is in general higher than normal cells mainly due to much higher expression and function of ion transporters contributing to the proton-transporting system [40,41,42,43]. Proteins in the cytosol bind with protons, buffering the intracellular pH. The bicarbonate (HCO_3_^−^) also reacts with proton), being protonated to H_2_CO_3_. Then, H_2_CO_3_ reaches equilibrium with the dissolved CO_2_ and H_2_O: $H^{+}+{\mathrm{HCO}}_{3}{}^{-}⇄H_{2}{\mathrm{CO}}_{3}⇄{\mathrm{CO}}_{2}+H_{2}O\;\left({\mathrm{pK}}_{a}=6.10\right)$. The main removing pathway of CO_2_ excess from the inside-to-outside of our bodies is the exhalation via the lung, participating in the removal of proton produced as CO_2_ at mitochondria under aerobic conditions or as lactic acid and/or ketone bodies such as β-hydroxybutyric acid consuming HCO_3_^−^. Although the bicarbonate–carbonate (HCO_3_^−^−CO_2_) system in general plays an important role in pH stabilization, MM and DM cells produce little or no CO_2_ inside of the cells due to mitochondrial dysfunction. Therefore, the bicarbonate–carbonate (HCO_3_^−^−CO_2_) system shows little or no contribution to pH stabilization in MM or DM cells based on the CO_2_ produced inside of the cells. However, metabolic cells, in general, express ion transporters participating in HCO_3_^-^ uptake into the intracellular space from the extracellular space such as Na^+^-HCO_3_^−^ cotransporter (NBC) and Na^+^-driven Cl^−^/HCO_3_^−^ exchanger (NDCBE) (Figure 1) [48]. Thus, although the bicarbonate–carbonate (HCO_3_^−^−CO_2_) system shows little or no contribution to pH stabilization in MM or DM cells based on no or little production of CO_2_ inside of the cells, some parts of the intracellular pH stabilization would be conducted by HCO_3_^-^ incorporated into the intracellular space from the extracellular one by NBC and NDCBE (Figure 1) [48]. In addition, phosphoric acid acts as a buffering factor: $H^{+}+{\mathrm{HPO}}_{4}{}^{3-}⇆{\mathrm{HPO}}_{4}{}^{2-}\;\left({\mathrm{pK}}_{a}=12.67\right)$; $H^{+}+{\mathrm{HPO}}_{4}{}^{2-}⇆H_{2}{\mathrm{PO}}_{4}{}^{-}\;\left({\mathrm{pK}}_{a}=7.21\right)$; $H^{+}+H_{2}{\mathrm{PO}}_{4}{}^{-}⇆H_{3}{\mathrm{PO}}_{4}\;\left({\mathrm{pK}}_{a}=2.12\right).$ Under physiological and pathophysiological pH ranges (6.0~7.6) in body fluids such as extracellular fluids including serum and intracellular fluids, phosphoric acid plays a role as a pH buffering system with the action $H^{+}+{\mathrm{HPO}}_{4}{}^{2-}⇆H_{2}{\mathrm{PO}}_{4}{}^{-}\;\left(\mathrm{pKa}=7.21\right)$ [48,49,68,69,70]. These pH buffering systems minimize the pH change caused by acids produced in metabolic cells in MM and DM.

### 6.3. The Extrusion System of Acids (Protons) from the Intracellular Fluid to the Extracellular One in MM and DM

Some of ion transporters participating in the extrusion system of acids (protons) from the intracellular fluid to the extracellular fluid actively contribute in creating and maintaining extracellular acidity in MM cells (see Section 5). In this section, we provide an overview on specific characteristics of the ion transporters involved in the intracellular pH stabilization. Various types of ion transporters contribute to protons excretion from the intracellular to the extracellular fluids, with the purpose to maintain the intracellular pH within normal ranges [48]. For example, monocarboxylate cotransporters (MCTs) extrude protons coupled with monocarboxylate anions, such as lactate, pyruvate, acetoacetate and β-hydroxybutyrate, metabolites in metabolic cells, across the plasma (cellular) membrane to the extracellular fluid [32,37,42,71,72,73]. Only MCT1, 2 and 4 out of 14 MCT isoforms are involved in β-hydroxybutyrate transport. MCTs are tissue-specifically expressed: MCT1 is ubiquitously expressed, but highly expressed in myocytes [74], adipocytes [41], and MM cells [40]; MCT2, specifically in the brain and the kidney but not in MM; MCT4, in skeletal muscle, adipocytes, the heart, the lung, and the brain [41,43,74,75], also in malignant tumor cells [76] including MM cells [40]. MCT11 has been reported to play a key role in extrusion of lactate to keep the normal intracellular pH [77]. Further, the NHE contributes to the release of protons from the intracellular fluid to the extracellular one: HCO_3_^−^-coupled transporters also contributes to reduction of proton concentration (with pH increase) in the intracellular space via incorporation of HCO_3_^−^ into intracellular space; via the carbonic anhydrase-accelerated process, $H^{+}+{\mathrm{HCO}}_{3}{}^{-}\to H_{2}{\mathrm{CO}}_{3}\to {\mathrm{CO}}_{2}+H_{2}O$, and H^+^-coupled transporters also play a role in extrusion of H^+^ from the intracellular space to the extracellular one [32,37,48]. Thus, protons produced in the intracellular space are extruded to the extracellular fluid for maintenance of the intracellular fluid pH by these ion transporters. The proton extrusion to the extracellular fluid from the intracellular one causes acidity in the extracellular fluid. In severe metabolic disorders including severe DM, systemic acidosis occurs with artery blood pH < 7.35. However, even in a case with normal arterial blood pH, DM notably shows acidic microenvironments with low pH (<7.35) around metabolic cells such as myocytes, adipocytes and neural cells in the brain similar to those around cancer cells [48,49,60,70,78,79,80,81,82,83,84]. The difference of pH values between microenvironments around metabolic cells and arterial blood is due to the pH buffer capacitance in both areas [48]: i.e., blood has strong pH buffer capacitance such as albumin and hemoglobin, while the interstitial fluid has little pH buffer capacitance [48,49].

DM patients produce acidic microenvironments similar to the acidity appearing at microenvironments of MM cells via the glycolysis-based metabolism. It is mandatory that the acidic microenvironment may represent a key pathogenetic mechanism predisposing DM patients to cancer development, through a huge amount of proton production and release in the extracellular environment at tissue levels. All in these observations suggest two possible clinical implications for MM: one is that MCTs inhibitors (MCTIs), similarly to PPIs may well be included in novel protocol of therapies against MM; the other implication is that MM patients may benefit of a hypocaloric diet in order to avoid the shift in a glycolysis-based metabolic process occurring in DM and thus favoring the generation of an acidic microenvironment in turn predisposing to MM at the bone level [83].

## 7. Conclusions

**Figure 2:**
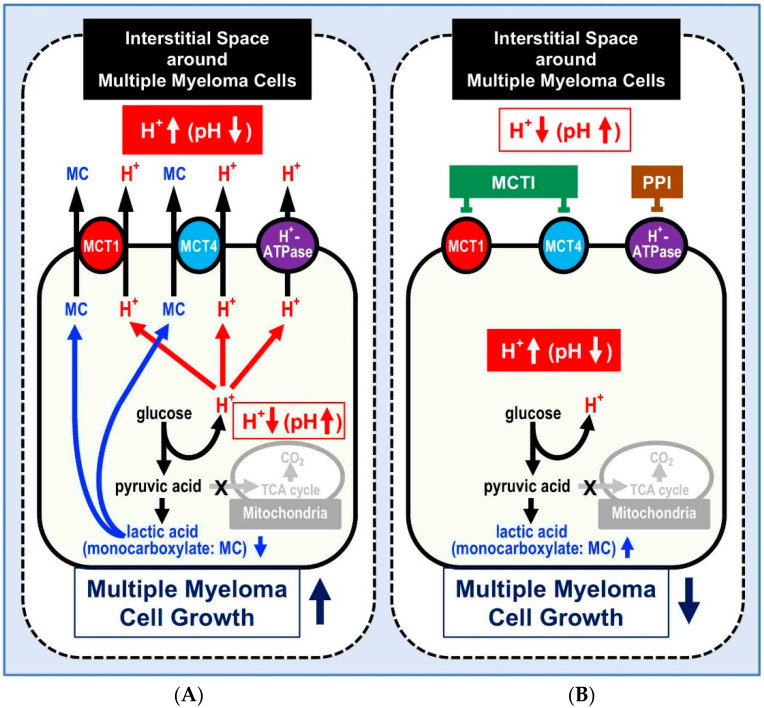
Production and transporting systems of H^+^ and lactic acid without (**A**) and with (**B**) inhibitors of proton pumps and/or MCT in multiple myeloma (MM) and diabetic mellitus cells with mitochondrial dysfunction under normoxemic or hypoxemic conditions. (**A**) Production and transporting systems of H^+^ and CO_2_ in normal cells with normal mitochondrial function under normoxemic conditions. H^+^ is extruded by H^+^-ATPase and MCT. Lactic acid is extruded by MCTs coupled with H^+^. (**B**) Production and transporting systems of H^+^ and CO_2_ in multiple myeloma (MM) and diabetic mellitus cells with mitochondrial dysfunction under normoxemic or hypoxemic conditions. MCT, Mono-Carboxylate Transporter: MCTI, Mono-Carboxylate Transporter Inhibitor: PPI, Proton Pump Inhibitor.

As we have introduced this review, multiple myeloma is unfortunately an unmet clinical problem, with an unbearable number of deaths worldwide [84,85,86,87]. It appears therefore mandatory to propose alternative treatment options for MM. This review has firstly proposed a new strategy that is not based on specific molecular targets identified in MM cells. Rather, an approach based on a feature common to all cancers, the extracellular acidic pH of tumor cells, in turn leading to the acidification of the whole tumor microenvironment [7,88]. Of course, this does not represent a mainstream approach to anti-cancer therapy, but many pre-clinical investigations and some clinical studies have supported the use of anti-acidic approaches as a valuable weapon against tumors. However, the only one approach that is directed against a cellular target is the use of proton pump inhibitors (PPIs). The target cellular target of proton pump inhibitors (PPI) are the vacuolar type H^+^-ATPases (V-type H^+^-ATPases), which have shown to exert a key role in the extracellular acidification of tumors through the extrusion of H^+^ protons. This review emphasizes the data obtained with a class of PPIs, which are used as anti-acidic drugs worldwide by hundreds of millions of people. Contrarily to all the other chemical molecules, this family of drugs are all administered as prodrugs, becoming the active molecule (tetracyclic sulfonamide) when arrived into the acidic tumor microenvironment and of course the intracellular acidic spaces. In few words, protonation while blocking the majority of weak bases outside the cells induces activation of these drugs, which in their active form, are able to block a wide panel of proton pumps including both V-type H^+^-ATPases and H^+^/K^+^ ATPase [7]. The inhibition of proton pumps involves a block of H^+^ traffic from the cytosol to both the internal vesicles and the extracellular spaces [10]. The experimental evidence has shown that, starting from the typical pH gradients reversal (Figure 2A), the PPI action leads to a very quick cell death, which is in fact due to an internal acidification of tumor cells (Figure 2B), but involves an atypical cell death that in MM cells is caspase independent [33]. Some unpublished data obtained with transmission electron microscopy (TEM) suggest that the PPIs induce cell death via a block of transcription in the treated tumor cells, and the nucleoli of the analyzed cells are entirely empty following the PPIs treatment. Thus, the unpublished data further provide the evidence that PPIs induce a non-conventional cell death in tumor cells. All PPIs have been successfully used in a wide panel of human cancers including hematological neoplasms, in both single and combined therapies [15,16,17,18,24,25,26,27]. However, PPIs have been shown to improve immune therapies as well, suggesting that they may have a systemic action [35]. The PPI Lansoprazole, is the only one successfully tested against MM cells, thus providing a novel therapeutic option for MM patients. PPIs, however, represent a model for future anti-tumor drugs, based on the evidence that this class of drugs should be re-thought as prodrugs targeting acidic spaces and there transformed into the active molecule following protonation. Further, we should consider another strategy against MM using MCTs inhibitors (MCTIs) expressed in MM, since MCTs contribute to keep the intracellular pH like H^+^-ATPase (Figure 2). The most important point from the viewpoint of usage of MCTIs as anti-MM drugs is that MCTIs can be the active form inhibiting MCTs only under the severe acidic condition around MM cells similar to PPIs.

This review includes also the analysis of the analogies between MM and DM, supporting the use of PPIs and MCTIs in MM patients but of a hypocaloric diet as well. It is however mandatory to use PPIs combination [30] and possibly new types of MCTIs in the treatment of both MM and DM.

## Abbreviations

ALL	acute lymphoblastic leukemia
DM	diabetes mellitus
MCTIs	monocarboxylate transporters inhibitors
MCTs	monocarboxylate transporters
MM	multiple myeloma
NBC	Na^+^-HCO_3_^-^ cotransporter
NDCBE	Na^+^-driven Cl^-^/HCO_3_^-^ exchanger
NHE	Na^+^/H^+^ exchanger
OS	overall survival
PFS	prolong progression-free survival
PI	proteasome inhibitor
PPIs	proton pump inhibitors
ROS	reactive oxygen species
TEM	transmission electron microscopy
V-type	vacuolar type
